# Uranium (VI) detection in groundwater using a gold nanoparticle/paper-based lateral flow device

**DOI:** 10.1038/s41598-018-34610-5

**Published:** 2018-11-01

**Authors:** Daniel Quesada-González, Grace A. Jairo, Robert C. Blake, Diane A. Blake, Arben Merkoçi

**Affiliations:** 1 Nanobioelectronics & Biosensors Group, Catalan Institute of Nanoscience and Nanotechnology (ICN2), CSIC and BIST, Campus UAB, Bellaterra, 08193 Barcelona, Spain; 2 Department of Biochemistry and Molecular Biology, Tulane University School of Medicine, 1430 Tulane Avenue, New Orleans, Louisiana 70112 United States; 3 Division of Basic Pharmaceutical Sciences, Xavier University of Louisiana, 1 Drexel Drive, New Orleans, Louisiana 70125 United States; 4 ICREA, Institució Catalana de Recerca i Estudis Avançats, Pg. Lluís Companys 23, 08010 Barcelona, Spain

## Abstract

The contamination in groundwater due to the presence of uranium is nowadays a subject of concern due to the severe health problems associated with renal failure, genotoxicity and cancer. The standard methods to detect uranium require time-consuming processes and expensive non-portable equipment, so these measurements are rarely performed in-field, which increases the time until water samples are analysed. Furthermore, the few portable methods available do not allow quantitative analysis and the detection limit is often not low enough to reach the recommendations for drinking water (30 ppb or 126 nM of uranium). For the first time, we propose a portable, fast, inexpensive and sensitive paper-based biosensor able to detect *in situ* U(VI) in water samples: U(VI) selective gold nanoparticle-based lateral flow strips. Antibody-coated gold nanoparticles are used as labels in the proposed lateral flow system because of their biocompatibility; in addition, these nanoparticles provide high sensitivity due to their intense plasmonic effect. The antibody used in the assay recognizes soluble U(VI) complexed to the chelator, 2,9-dicarboxyl-1,10-phenanthroline (DCP). Because of the small size of the U(VI)-DCP complex, this assay employs a competitive format that reaches a limit of detection of 36.38 nM, lower than the action level (126 nM) established by the World Health Organization and the U.S. Environmental Protection Agency for drinking waters.

## Introduction

Uranium enters the environment from mining and ore processing^1,2^ and the extensive use of phosphate fertilizers^3,4^. The uranium that exists naturally in granite and other mineral deposits^1^ can also contribute to groundwater contamination. Uranium contamination in both ground and surface waters have also significantly increased in the last two decades due to military use of the depleted metal^5,6^. In groundwater, this heavy metal is most commonly found in its hexavalent form, U(VI), also referred to as uranyl ion (UO_2_^2+^). The current maximum contamination level for uranium in drinking water, as stipulated by the U.S. Environmental Protection Agency^7^ (EPA) and the World Health Organization^1^(WHO) is 30 µg/L (126 nM). The consumption of high amounts of uranium has been associated with renal problems (accumulation in kidney), genotoxicity and cancer development (e.g. leukaemia due uranium accumulation in bones)^8,9^, among others. Current detection methods for uranium, such as ICP-MS (inductively coupled plasma mass spectrometry)^10^, kinetic phosphorescence analysis^11^ or radiation detection systems^12^, require several sample extraction and pretreatment steps, expensive equipment, and highly trained personnel; such analyses are mainly performed in high-tech laboratory settings. Although there exist portable radiation detection equipment, the detection is mostly qualitative and only possible for high amounts of the radioactive isotopes^12^.

It has been the long-term interest of environmental scientists to seek new methods to rapidly detect and quantify water contaminants at the site of contamination. In recent years, functional nucleic acids, also called DNAzymes, have been selected from random nucleic acid libraries containing a very large number of sequences (>10^14^) for the rapid recognition small molecules, including Pb(II), Mg(II), Zn(I) and Cd(II)^13–16^. The metal ion-specific DNAzymes are minimally fluorescent in the absence of the metal ion because the fluorophores in the hybridized DNA strands are quenched by proximity to a quencher. In the presence of a specific metal ion, the DNA is cleaved into two pieces, which results in an increase in fluorescence proportional to the metal ion concentration^17^. This dependence upon metal ion concentration is consistent with the published mechanism of the cleavage reaction, which involves a metal-assisted deprotonation of the substrate nucleic acid^18^. While this method is highly reliable in simple solutions, it is subject to a variety of interferences in environmental water samples, because in naturally occurring water samples, metals are almost always complexed to other components of the environmental matrix, including humic and fulvic acids^19,20^. These interferences are further complicated in the analysis of (U(VI)), which also forms tight bi-tri- and tetradentant complexes with calcium, carbonate, and phosphate in addition to organic materials^21–25^. Thus, the state of U(VI) complexation can vary widely depending upon the conditions at difference environmental sites, and such complexation can influence the ability of the metal ion to participate in the cleavage reaction required to generate a signal in the DNAzyme-based assays.

In our laboratories, we have approached the problem of the complexation of metal ions in natural waters by selecting monoclonal and recombinant antibodies that bind tightly to metal-chelate complexes, but much less tightly to the metal-free chelator^26–29^. Environmental and/or clinical samples are acidified to release the metal ion from its complexant(s) and then neutralized in the presence of a molar excess of chelator, which transforms the metal to a metal-chelate complex that is recognized by the antibody. This simple pretreatment procedure has resulted in ELISA and sensor-based immunoassays that agree with instrumental analyses^30–33^.

Herein, we report the development of lateral flow strips (LFs) for the analysis of U(VI). LFs are paper-based immunosensors that can be used to detect the presence of specific molecules in a given sample. These devices are simple, portable, cheap to produce, and do not require complicated sensors or highly skilled labor^34–37^. The use of LFs has previously been reported for the detection of other heavy metals such as lead^38^, cadmium^39^ and mercury^40^. In the present study, a monoclonal antibody (clone 12F6) that specifically recognizes UO_2_^2+^ complexed to the chelator, 2,9-dicarboxyl-1,10-phenanthroline (DCP) was used for the analysis^27^. This chelator binds to UO_2_^2+^ with an affinity five orders of magnitude greater than that of conventional metal chelators such as ethylenediaminetetraacetic acid (EDTA)^27^. The integration of nanoparticles^36^ on LFs leads to higher sensitivity and lower detection limits in comparison to other materials used as labels. Gold nanoparticles (AuNPs) were chosen as labels in this assay due to their strong red color and biocompatibility with antibodies^41–44^.

## Materials and Methods

### Materials

The 12F6 antibody was prepared and characterized as previously described^27,32,45^. Bovine serum albumin (BSA), goat anti-mouse IgG polyclonal antibody, tetrachloroauric acid (HAuCl_4_), trisodium citrate, Tween 20, sucrose and the reagents used to prepare HEPES-buffered saline (HBS: NaCl 137 mM, KCl 3 mM and HEPES 10 mM; pH 7.4) were purchased from Sigma Aldrich. Analytical grade nitric acid was purchased from Fisher Scientific. The U(VI) stock solution was purchased from Perkin Elmer and the chelator, 2,9-dicarboxyl-1,10-phenanthroline (DCP) was purchased from Alpha Aesar. Cellulose membranes (CFSP001700), glass fiber (GFCP00080000), nitrocellulose membranes (HF180), and adhesive laminated cards were purchased from Millipore.

### Synthesis of gold nanoparticles (AuNPs)

AuNPs of approximately 20 nm diameter were synthesized by citrate reduction of HAuCl_4_ following the Turkevich method^46^. A 0.01% (w/v) solution of HAuCl_4_ was prepared by diluting 0.5 ml of a 1% HAuCl_4_ to a final volume of 50 mL in Milli-Q water. This solution was heated to boiling point and 1.25 mL of 1% (w/v) sodium citrate was added under continuous vigorous stirring. The solution was allowed to boil for an additional 10 minutes then allowed to cool to room temperature. The AuNP solution was adjusted to a pH of 9.0 by addition of 0.01 mM borate buffer. The AuNPs were characterized by transmission electron microscopy (TEM, Fig. S1).

### Preparation of the 12F6-AuNP conjugate

An aliquot (200 μg/mL in HBS, 100 µL) of the 12F6 antibody was added to 1.5 mL of AuNP solution and incubated for 20 minutes at room temperature. BSA solution (5% (w/v), 100 µL) was then added and the mixture was allowed to incubate for another 20 minutes followed by centrifugation at 4 °C (14000 rpm, 20 minutes). The supernatant was discarded and the pellet was reconstituted in 0.5 mL HBS buffer containing 10% sucrose. The ratio antibody:AuNP was calculated to be 7:1, as explained in supporting information.

### Preparation of the U(VI)-DCP-bovine serum albumin conjugate

The DCP-bovine serum conjugate was prepared by incubating BSA at a 1:60 molar ratio with 5-isothiocyanato-1,10-phenanthroline-2,9-dicarboxylic acid (synthesized as described in ref.^27^) in 0.1 mM HEPES, pH 9.0, at 25 °C overnight, then dialysing the reaction mixture in HBS. This procedure results in covalent conjugation of the DCP to the lysines of BSA via a thioether linkage. The conjugate concentration was determined using the BCA protein assay (Fisher Scientific). The DCP-BSA conjugate was diluted to a concentration of <100 µg/mL in HBS and pre-loaded with U(VI) by addition of a 1.1 molar excess of uranyl acetate. The solution was incubated on an end-over-end rotator for 1 hour and any unbound uranyl acetate was removed using a PD-10 desalting column (GE Healthcare).

### Preparation of the lateral flow strips

The sample pad was prepared by dipping the cellulose membrane in HBS buffer containing 5% BSA and 0.05% Tween 20. The membrane was dried at 60 °C for 2 hours and stored in barrier zip-lock pouches with drying pearls at 4 °C. The conjugate pad was prepared by dispensing freshly prepared AuNP-antibody conjugate suspension onto the glass fiber. The fiber was dried by quickly transferring to a vacuum chamber for 2.5 hours, and then stored in barrier zip-lock pouches with drying pearls at 4 °C. The detection pad consists of a nitrocellulose membrane, onto which proteins can be bound^47–49^, assembled onto an adhesive laminated card backing. Using the Biofluidix Biospot Workstation, approximately 1 μL/mm of the U(VI)-DCP-BSA conjugate (0.25 mg/mL) and goat anti-mouse IgG polyclonal antibody (1.00 mg/mL) solutions were dispensed onto the test line (TL) and the control line (CL) of the detection pad, respectively. The detection pad was dried at 37 °C for 2 hours and stored at room temperature under dry conditions.

The conjugate pad was assembled onto the adhesive backing with a 1 mm overlap over the detection pad. The sample pad was assembled overlapping the conjugate pad at the end of the strip. On the other end, an absorption pad (untreated cellulose membrane) was assembled overlapping the detection pad, at 3 mm away from the CL. The strips were cut to a width of 6 mm using a guillotine and stored at room temperature in dry conditions.

### Assay performance

Standard U(VI) solutions (25 nM to 800 nM) were prepared by diluting the uranium stock solution (1000 ppm; 4.2 mM) in HBS. An aliquot (100 μL) of each U(VI) sample was mixed with 100 μL of 2 μM DCP solution and after incubation for 10 minutes at room temperature, the samples were applied on the sample pad of the strips. After 5 minutes, TL and CL were observed. After 20 minutes, images of the strips were taken using a mobile phone camera^50–53^ (Moto Z, 13 megapixel). Image J software was used to quantify the color intensities of the TLs. The images were converted to 8-bit black-and-white format and the intensities of white pixels were measured on the TL, with an intensity value of 255 considered pure white while an intensity value of 0.0, pure black. Data was normalized into % color where 0% and 100% equal to 255 and 0 values respectively.

### Evaluation of strips with contaminated groundwater samples

Uranium-contaminated groundwater samples were obtained from a uranium contaminated site in Rifle, Colorado (Fig. S2)^27^. These groundwater samples were filtered through a 0.45-micron non-uranium binding filter to remove any particulate matter before they were received at Tulane University. To prevent the precipitation of uranium, the samples were acidified to a pH of 2.0 by adding 125 µL of 8 M nitric acid per 50 mL of sample. Because the binding of the 12F6 antibody to the U(VI)-DCP complex is inhibited by mM quantities of ionic calcium (see Fig. S3 in Supporting Information), standard curves for groundwater samples were therefore adjusted to have the same [Ca^2+^] as the groundwater sample. This was achieved by treating a small quantity of the contaminated water sample with uranium absorbing particles that completely remove the U(VI) but not the Ca^2+^ from the groundwater^54^. The Ca^2+^ concentration in the standards and the samples could then be normalized by keeping the final concentration of groundwater (treated or untreated) at 10% in all samples and standards. A detailed explanation of the uranium absorbing particles and the method for removing U(VI) from the groundwater samples is available in Supporting Information. Tables S1, S2 in Supporting Information detail the preparation of the standards and samples incorporating the groundwater. The pH of the samples and the standards were checked before they were applied to the strips.

## Results and Discussion

### Design of the AuNP-based LFs for the detection of U(VI)

This lateral flow assay relies on the 12F6 monoclonal antibody’s specific recognition of the U(VI)-DCP complex. The metal ion specificity of this antibody has been described briefly in a previous report^27^. Table S1 in Supporting information provides additional data about of 12F6 cross reactivity with >20 different mono-, di-, tri-, tetra- and hexavalent metal ions tested in low µM concentrations. Under these conditions, the 12F6 antibody is remarkably specific for the DCP-U(VI) complex. The highest degree of cross reactivity was with Cu^2+^, but the cross reactivity was only 0.0206%. Hg^2+^, Al^3+^ and Mb^6+^ also showed measurable cross reactivity (0.0153–0.005%) and all other metals tested (Ag^1+^, Au^3+^, Ca^2+^, Cd^2+^, Co^2+^, Er^3+^, Fe^3+^, In^3+^, Mg^2+^, Mn^2+^, Ni^2+^, Pb^2+^, Pr^3+^, Sr^2+^, Ti^3+^, Y^3+^ and Zn^2+^) had cross reactivity of less than 0.0.0033%. At 1000-fold higher concentrations than those tested in this analysis (2–85 mM), calcium inhibited the binding to the immobilized U(VI)-DCP-BSA conjugate, most likely by changing the conformation of the antibody molecule (see below and Supporting Information).

**Figure 1:**
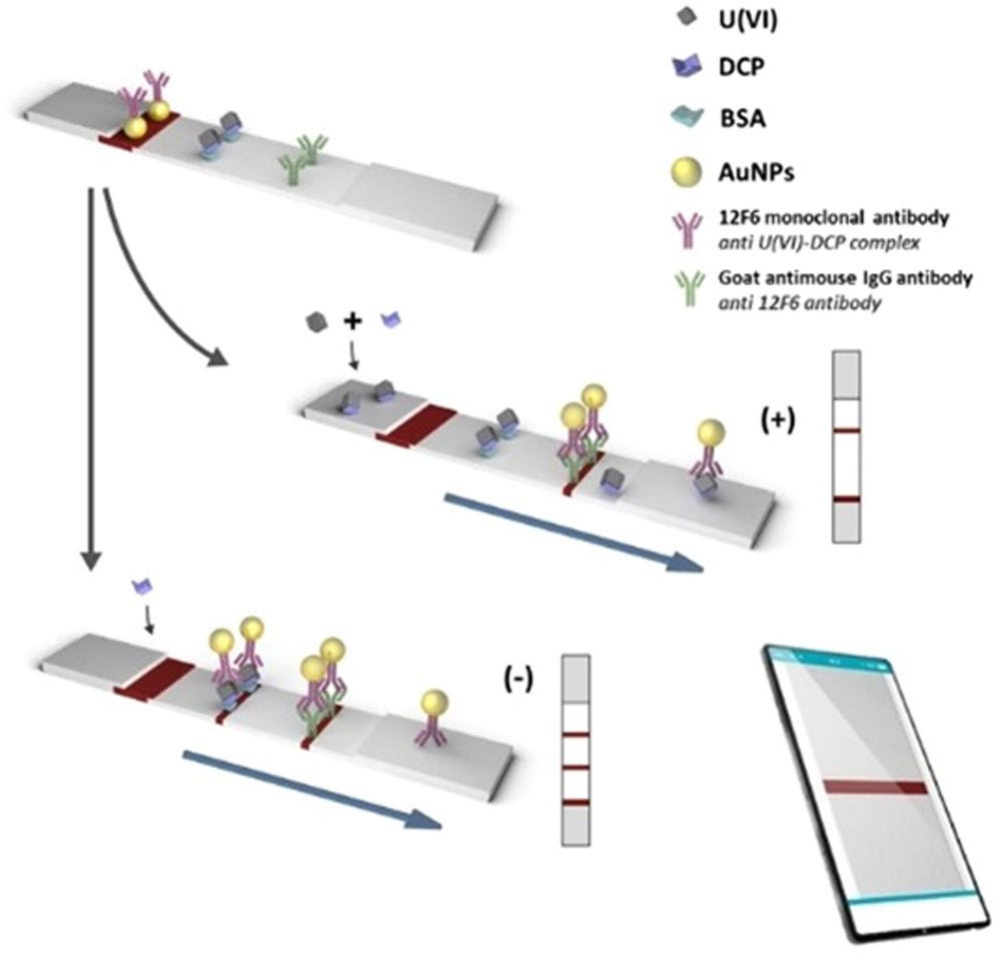
Schematic (not drawn to scale) of the configuration and operating principle of the lateral flow strip for the detection of U(VI). The intensity on the strips can be measured by using a mobile phone camera.

Due to the small size of the chelated uranium, it is not possible to form the typical “capture antibody-analyte-recognition antibody” immunosandwich on the TL, as seen with most standard LFs models^36^. Therefore, this immunoassay was designed based on a competitive model^39^ as illustrated in Fig. 1. This results in less red coloration on the TL as the U(VI) concentration in the sample increases. In both the positive and negative control assays, the excess AuNP-12F6 complex is captured on the CL by an immobilized goat anti-mouse antibody, resulting in an intense red line. Thus, in a negative assay (−), both the TL and CL should have an intense red coloration. Increasing concentrations of U(VI) in the sample should result in a decrease in the intensity of the TL. The color intensity of the TL is thus inversely proportional to the concentration of U(VI) in the sample.

### Optimization of assay conditions

**Figure 2:**
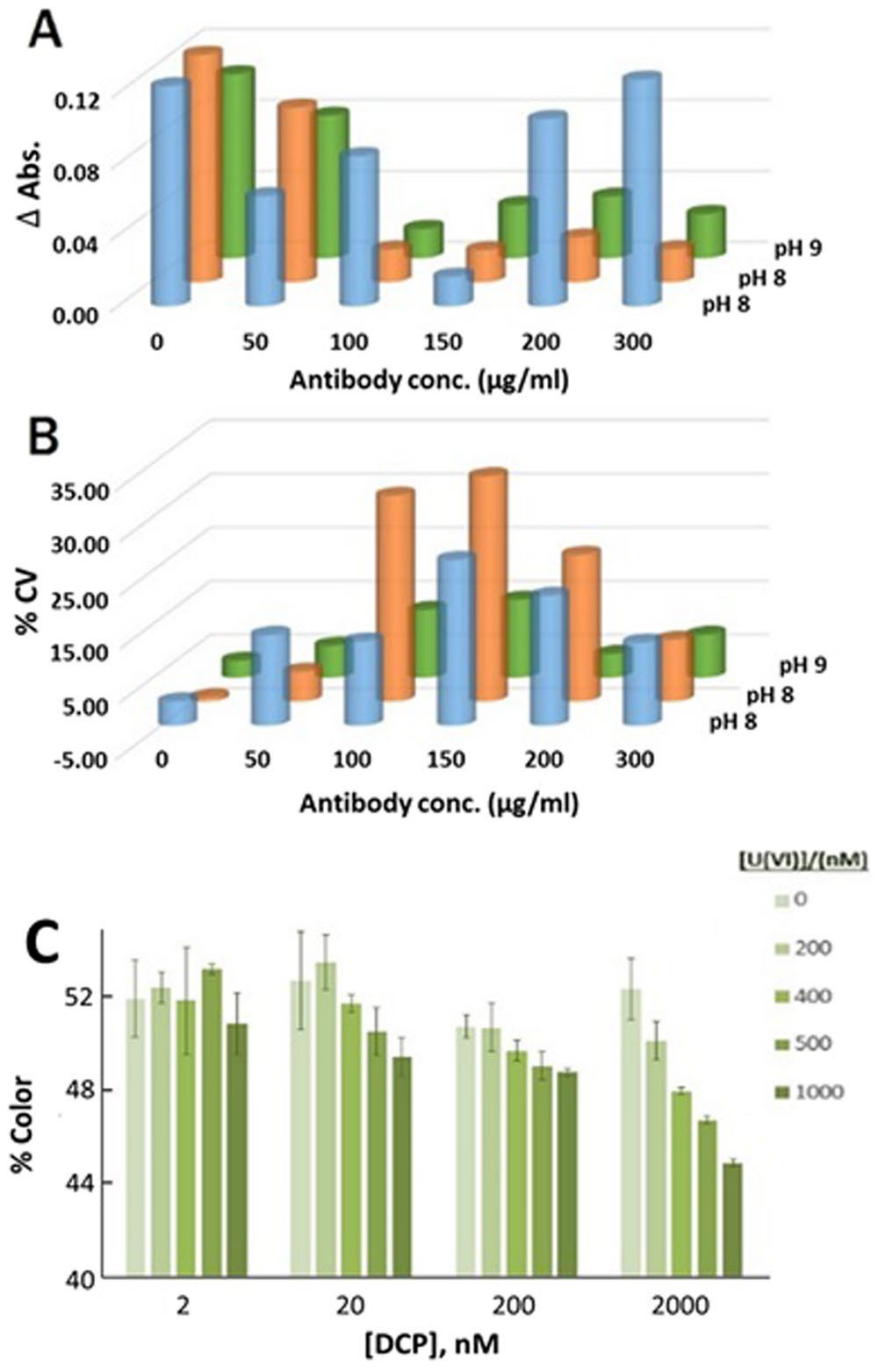
(**A**) Evaluation of the conjugation of 12F6 antibody at different concentrations on AuNPs at pH 7, 8 and 9 by means of the variation of absorbance (ΔAbs.) caused by the addition of NaCl and (**B**) the corresponding coefficient of variation (CV, %) of each point. (**C**) Evaluation of the linearity of the color decrease related to U(VI) concentration for LFs at different concentrations of DCP.

It was necessary to optimize various assay conditions in order to ensure proper functionality of the LFs. First, a gold nanoparticle aggregation test^55^ was performed to determine the optimal concentration of the 12F6 antibody that could be conjugated onto AuNPs. AuNP suspensions at various pH values (7, 8, and 9) were mixed with varying concentrations (0, 50, 100, 150, 200, and 300 µg/mL) of the 12F6 antibody. The UV-Vis absorbance of these solutions at 520 nm were measured before and after addition of NaCl, using a Spectra Max iD3 spectrophotometer. If the amount of antibody was not enough to cover the surface of the AuNPs, the presence of the NaCl in solution would cause the AuNPs to aggregate and subsequently precipitate out of solution. Figure 2A shows the absorbance values recorded at various pH values with different concentrations of antibody and Fig. 2B shows the corresponding % CV values of the absorbance values reported in Fig. 2A. Based on this data, a pH 9.0 and antibody concentration of 200 μg/mL were chosen for the conjugation.

In this assay, sample pre-treatment with the chelator, DCP, was required before dispensing onto the LFs. Various concentrations (2, 20, 200 and 2000 nM) of DCP were mixed with different concentrations (0, 200, 400, 500 and 1000 nM) of U(VI), and applied onto the LFs. The DCP and U(VI) solutions were mixed at a 1:1 ratio (by volume) and allowed to incubate for 10 minutes at room temperature. 200 µL of each mixture were applied onto the LFs. Images of the strips were taken and color intensities of the TL were quantified using ImageJ software. Figure 2C shows the % color observed from these experiments. Based on these data, a DCP concentration of 2000 nM was chosen for the rest of the assays since higher sensitivity and linear behaviour were observed.

**Figure 3:**
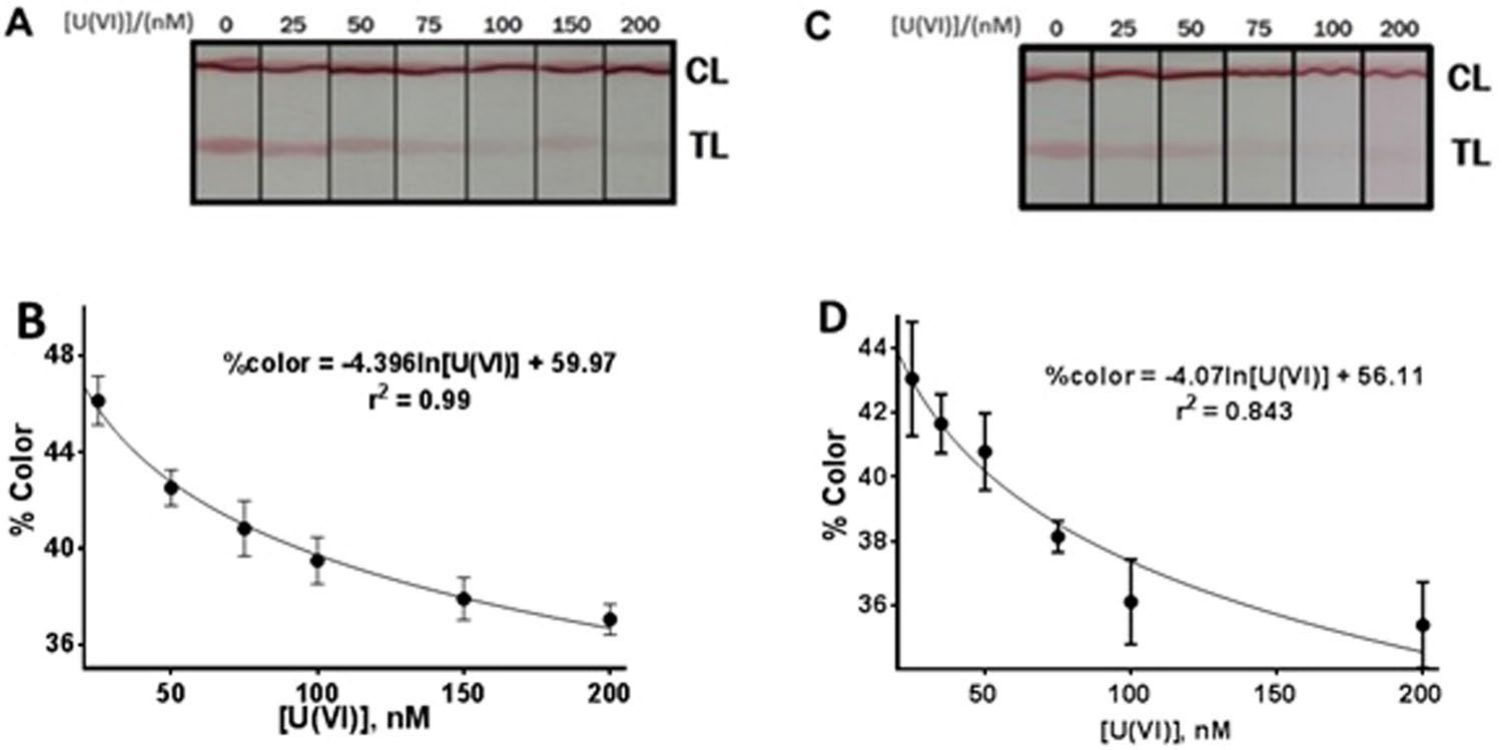
(**A**) LFs response at U(VI) concentrations between 0 and 200 nM in HBS buffer and (**B**) the corresponding working range equation. The data shown in (**B**) was collected from two different experiments, performed one month apart, with two different LF strip batches. The color intensities at each U(VI) concentration were measured in triplicate. (**C**) LFs response at various standard U(VI) concentrations in HBS buffer supplemented with pre-treated groundwater and (**D**) the corresponding working range equation. This curve was generated from two experiments approximately one month apart in which % color at each concentration was measured in duplicates (n = 4).

Samples containing various concentrations of U(VI) standards were prepared as described in the methods section and evaluated with the LFs to determine the optimal working range of the assay. All samples and standards were prepared in HBS buffer, pH 7.4. Figure 3A is a representative image of the LFs and Fig. 3B shows the mathematical fit of the data obtained from the standards following the equation:

$$ \% \,{\rm{color}}=-\,4,389\,\mathrm{ln}\,[{\rm{U}}({\rm{VI}})]+59,895$$

The r^2^ for this fit was 0.9952 and the theoretical LOD (limit of detection) was calculated by replacing “% color” in the equation with the average signal of blank, 49%, plus 3 times its standard deviation, ±1%, resulting in a value of 6 nM. The method shows a reproducibility (RSD) of 2% (n = 6) for a 75 nM concentration of U(VI).

### Detection and quantification of U(VI) in contaminated groundwater samples

Since this LFs assay relies on the 12F6 antibody’s ability to recognize U(VI) in a complex with DCP, it was important to ensure that all the uranium in the environmental sample be dissociated from the various natural complexants^56^ in the groundwater before being complexed with DCP. To ensure dissociation of U(VI) from these natural complexants, the filtered groundwater samples were treated with 8 M nitric acid to lower the pH to ~2.0. This pre-treatment strategy was found to be the most successful in a previous study^32^, and the acidified samples could be stored for several months at 4 °C. A portion of the acidified environmental samples was sent for analysis by ICP-MS (Table S4 in Supporting Information). Samples were neutralized before applying onto the strips by mixing with HBS buffer containing DCP.

It has been previously observed that the very high concentrations (mM) of calcium in some groundwater samples inhibit the binding activity of the 12F6 antibody (see Fig. S2 in Supporting Information). The environmental samples in the present study contained ~7 mM Ca^2+^, and the presence of this much calcium in the groundwater samples could cause false positives in the results if the calcium concentration is not normalized in both the standards and the samples. To address this issue, uranium-contaminated groundwater samples were pre-treated with previously-described uranium absorbing particles^54^. These particles rapidly (within 5–10 minutes) and selectively remove uranyl ions but not calcium from groundwater samples. This treated groundwater can then be used both to prepare U(VI) calibrators for the test strips and, if necessary, to dilute environmental samples that contain concentrations of U(VI) higher than the concentration range of the LFs. These particles (see Fig. S4 in Supporting Information), were specifically developed for this immunoassay^54^ and make it possible to normalize the Ca^2+^ concentrations in the calibrators and samples on-site, without needing to know the actual Ca^2+^ in individual environmental samples (see Fig. S5 in Supporting information for schematic of environmental water handling).

Up to 98.7% of the uranium in the contaminated groundwater sample was removed by particle treatment (as quantified by ICP-MS, Table S3 in Supporting Information), and this pre-treated, U(VI)-free groundwater was used to supplement all the new standard solutions (10% pre-treated Rifle groundwater was added to each standard). This ensured that both the standards and the environmental samples were in the same background matrix. Samples were prepared by diluting the untreated acidified groundwater into HBS buffer containing DCP. The pre-treated groundwater was then used to adjust the volumes to ensure that each assay mixture applied to the sample pad contained 10% groundwater.

Three dilutions (10-, 15-, and 20-fold) of the groundwater sample were assessed. The pH of all the standards and samples applied to the lateral flow strips ranged between 7.2 and 7.4. Each standard and sample was assayed in quadruplicate. A representative image of the LFs used to generate standard curves with standards containing 10% pre-treated groundwater is shown in Fig. 3C. Figure 3D shows the standard curve generated from these LFs. The standard curve fits to the equation:

$$ \% \,{\rm{color}}=-\,4.07\,\mathrm{ln}\,[{\rm{U}}({\rm{VI}})]+56.11$$

The r^2^ value for the equation was 0.843 and the LOD 36.38 nM (the color intensity of blank plus 3 times its standard deviation.

U(VI) concentrations in the diluted groundwater samples were interpolated from the standard curve and the results are shown in Table 1. At a 15-fold dilution of the environmental sample, the measured U(VI) concentration was 51.70 nM, similar to the concentration measured by ICP-MS (48.23). However, larger differences were observed at 10-fold and 20-fold dilution factors, with the largest error at 20-fold dilution. The U(VI) concentration at 20-fold dilution is lower than the LOD and therefore a larger error was expected.

**Table 1:** Evaluation of environmental groundwater samples. Comparison of LFs and ICP-MS.

Environmental sample dilution factor	% colour	[U(VI)], nM, interpolated from the standard curve	[U(VI)], nM, by ICP-MS
10	39.55	58.50 ± 3.11	68.90
15	40.05	51.70 ± 2.94	48.23
20	40.17	50.21 ± 2.90	34.45

## Conclusions

A lateral flow assay for the detection and quantification of uranium in groundwater, using the 12F6 monoclonal antibody labelled with AuNPs as signal producer, has been developed for the first time. The 12F6 antibody has a primary specificity for uranyl ions complexed with the chelator, DCP. A competitive immunoassay format was used due to the small size of the analyte. Pictures of these lateral flow strips could be taken 20 minutes after adding sample and the color intensities were analysed using ImageJ software.

The strips exhibited a LOD of 6 nM when standards and samples were prepared in HBS buffer. When samples and standards were prepared in HBS buffer supplemented with 10% pre-treated groundwater, a LOD of 36.38 nM was observed. Both limits of detection are well below 126 nM, the maximum contaminant level for uranium in drinking water stipulated by the WHO and the United States EPA.

Pre-treating an aliquot of the contaminated groundwater with uranium absorbing particles provided a way to normalize for the calcium ion concentrations in the standards and samples. In this work, the particles were separated from the water by centrifugation or filtration. These particles can be further modified to contain a magnetic ferrite core. The particles can then be separated from the water using a simple magnet (available in most labs and portable). Thus, additional work/experiments are in progress to synthesize and characterize particles with a ferrite core^57^.

These lateral flow strips provide a potentially simpler and cheaper alternative to the current uranium detection and quantification methods. The assays can be performed *in situ* and provide results in less than 20 minutes. These strips can be used for both qualitative and quantitative analysis by combining the readout with a mobile phone application system.

## Electronic supplementary material

Supporting information
