# Supplementary material for: Uranium (VI) detection in groundwater using a gold nanoparticle/paper-based lateral flow device
Source: Sci Rep. 2018 Nov 1;8:16157. doi: 10.1038/s41598-018-34610-5 (PMC6212437; doi:10.1038/s41598-018-34610-5)
Supplement: Supplementary file 1 — Supporting information [file 41598_2018_34610_MOESM1_ESM.docx]

**Uranium (VI) detection in groundwater using a gold nanoparticle/paper-based lateral flow device**

**Daniel Quesada-González^1+^, Grace A. Jairo^2+^, Robert C. Blake II^3^, Diane A. Blake^2^ and Arben Merkoçi*^1,4^**

1 Nanobioelectronics & Biosensors Group, Catalan Institute of Nanoscience and Nanotechnology (ICN2), CSIC and BIST, Campus UAB, Bellaterra, 08193 Barcelona, Spain

2 Department of Biochemistry and Molecular Biology, Tulane University School of Medicine, 1430 Tulane Avenue, New Orleans, Louisiana 70112, United States Address here.

3 Division of Basic Pharmaceutical Sciences, Xavier University of Louisiana, 1 Drexel Drive, New Orleans, Louisiana, 70125, United States

4 ICREA, Institució Catalana de Recerca i Estudis Avançats, Pg. Lluís Companys 23, 08010 Barcelona, Spain

*arben.merkoci@icn2.cat

+These authors contributed equally to this work

SUPPORTING INFORMATION

Calculation of the ratio antibody:nanoparticle

The ratio antibody:nanoparticle was estimated dividing the surface area of a nanoparticle by the area occupied by an antibody, obtaining a rate of 7:1.

The surface area (A_1_) of a spherical nanoparticle was calculated as 1258 nm^2^ by the formula A_1_ = 4πr_1_^2^, being the radius (r_1_) of 10 nm. Then, the area occupied by an IgG molecule was estimated considering that the antibody occupies the area of a circle, being the radius of 7.5 nm.^1^ The formula applied was A_2_ = πr_2_^2^, obtaining an area of 176.71 nm^2^.

Figure S1. AuNPs characterized by transmission electron microscopy (TEM).


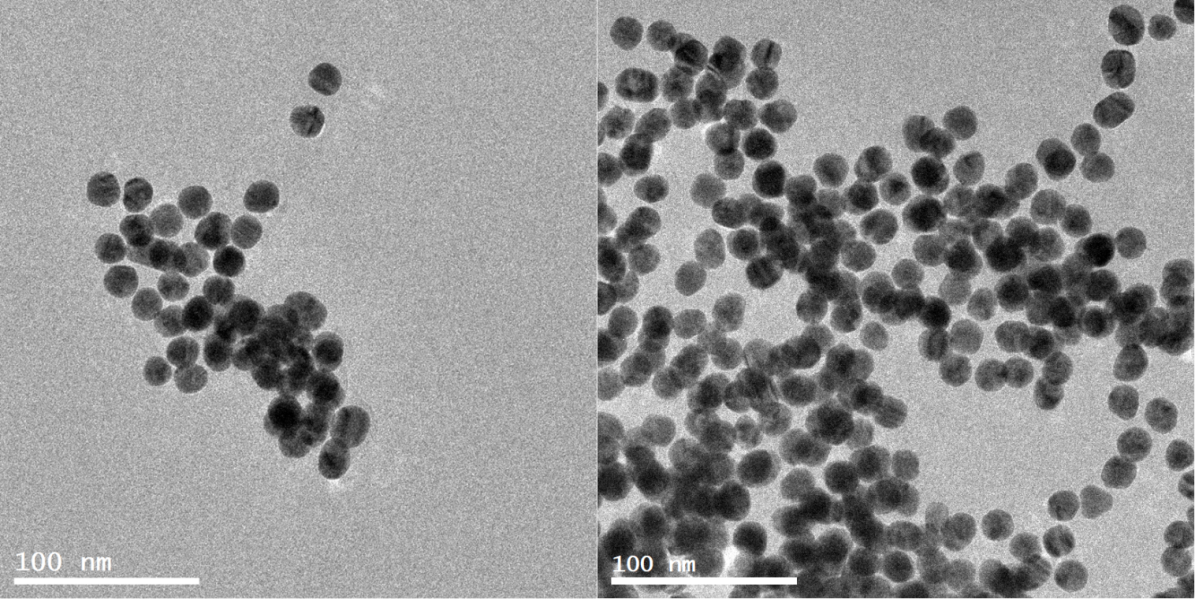


**
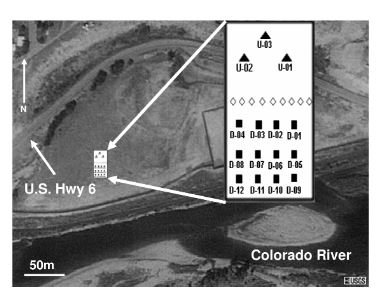
Figure S2.** Site of collection of uranium-contaminated groundwater

# The United States Department of Energy monitors a number of sites of former uranium mine/ore processing plants as part of the Uranium Mill Tailings Remedial Action (UMTRA) project. Groundwater for these studies was collected from a site located in Rifle, Colorado. The water used in these analyses was collected from one of the wells indicated by the ■ symbol. Reprinted with permission from ref 2: Melton, S. J., Yu, H., Williams, K. H., Morris, S. A., Long, P. E. & Blake, D. A. Field-Based Detection and Monitoring of Uranium in Contaminated Groundwater using Two Immunosensors. *Environ. Sci. Technol.* 43(17), 6703-6709 (2009)*.* Copyright 2009 American Chemical Society.



**Figure S3.** Effect of [Ca^2+^] on the binding of 12F6 to immobilized U(VI)-DCP-BSA

The effect of calcium on 12F6 binding was studied using a KinExA 3000 instrument and beads coated with U(VI)-DCP-BSA conjugate, as described previously.^3^ HBS was amended with CaCl_2_ at the indicated concentrations. 12F6 binding was inhibited by Ca^2+^in a dose-dependent fashion, the K_i_ for Ca^2+^ was 13+1 mM. These data were fit to the equation: y=a-b*x/(c+x), where a=maximum y value, b=difference between maximum and minimum y value, and c=K_i_. The r^2^ for the curve fit was 0.994.

**Figure S4.** SEM image of freshly-synthesized U(VI) absorbing particles.





Acrylonitrile-based uranium absorbing particles were synthesized and characterized using a previously described method^4^. Unlike other commericialy available resins, which can take up to 30 minutes these highly-porous particles were able to remove uranyl ions from goundwater samples within 5-10 minutes. The amidoxime (RC(=NOH)NH2) groups on the surface of these particles facilitate rapid and selective uptake of uranyl ions.

**Figure S5.** Schematic of environmental water handling

**Table S1.** Cross reactivity of 12F6 antibody with 21 different metal cations

Metal ions were mixed with DCP to generate solutions in HBS that contained 2 µM each of the metal ion and DCP. The samples were subsequently analyzed on the KinExA 3000 for their ability to inhibit the binding of the 12F6 antibody to immobilized U(VI)-DCP-BSA (described in more detail in ref 5, below). Percent cross reactivity was calculated as (IC_50_ of UO_2_^2+^/IC_50_ of tested metal) × 100.

| **Metal Ion** | **IC_50_ (µM)** | **% Cross Reactivity** |
| --- | --- | --- |
| UO_2_^2+^ | 0.00167 | 100 |
| Cu^2+^ | 8.1 | 0.0206 |
| Hg^2+^ | 10.9 | 0.0153 |
| Al^3+^ | 28.2 | 0.0059 |
| Mb^6+^ | 33.2 | 0.0050 |
| Ag^1+^ | >50 | <0.0033 |
| Au^3+^ | >50 | <0.0033 |
| Ca^2+^ | >50 | <0.0033 |
| Cd^2+^ | >50 | <0.0033 |
| Co^2+^ | >50 | <0.0033 |
| Er^3+^ | >50 | <0.0033 |
| Fe^3+^ | >50 | <0.0033 |
| In^3+^ | >50 | <0.0033 |
| Mg^2+^ | >50 | <0.0033 |
| Mn^2+^ | >50 | <0.0033 |
| Ni^2+^ | >50 | <0.0033 |
| Pb^2+^ | >50 | <0.0033 |
| Pr^3+^ | >50 | <0.0033 |
| Sr^2+^ | >50 | <0.0033 |
| Ti^3+^ | >50 | <0.0033 |
| Y^3+^ | >50 | <0.0033 |
| Zn^2+^ | >50 | <0.0033 |

**Table S2.** Preparation of standards containing pre-treated groundwater samples

All standard solutions were prepared such that each prep contained a final concentration 1µM DCP, and each sample also contained 10% of pre-treated Rifle groundwater. All final solutions had a pH of 7.2-7.4.

200 µL (per strip) of each standard solution was dispensed onto the LFs.

| [UO_2_^2+^] (nM): | **0** | **12.5** | **25** | **35** | **50** | **75** | **100** | **200** |
| --- | --- | --- | --- | --- | --- | --- | --- | --- |
| Vol. of stock (2 µM U(VI), in HBS) (µL) | 0 | 3.13 | 6.25 | 8.75 | 12.5 | 18.75 | 25 | 50 |
| Pre-treated Rifle groundwater (µL) | 50 | 50 | 50 | 50 | 50 | 50 | 50 | 50 |
| HBS (µL) | 200 | 196.87 | 193.75 | 191.25 | 187.5 | 181.25 | 175 | 150 |
| 2000 nM DCP in HBS (µL) | 250 | 250 | 250 | 250 | 250 | 250 | 250 | 250 |
| Total Vol. (µL) | 500 | 500 | 500 | 500 | 500 | 500 | 500 | 500 |

**Table S3.** Preparation of environmental samples with U(VI) concentrations.

Three different dilutions (10, 15 and 20-fold) of the acidified environmental samples were prepared for analysis following the table below. Pre-treated groundwater was used to supplement such that all the final prepared samples contained 10% Rifle groundwater. All final solutions had a pH of 7.2-7.4 and 200 µL of each (per strip) was dispensed onto the LFs.

| **Sample dilution factor** | **10fold** | **~15fold** | **20fold** |
| --- | --- | --- | --- |
| Vol. of Rifle groundwater (Acidified) (µL) | 50 | 35 | 25 |
| Pretreated Rifle groundwater (Neutralized) | 0 | 15 | 25 |
| HBS | 200 | 200 | 200 |
| 2000 nM DCP in HBS | 250 | 250 | 250 |
| Total Vol. (µl) | 500 | 500 | 500 |

**Table S4.** Concentration of uranium and calcium in untreated groundwater and groundwater treated with uranium absorbing particles. Metal concentrations were determined using a Thermo Element2 ICP-MS at the Tulane Coordinated Instrument Facility.

|  | **Untreated** | **Treated** | **% Removed** |
| --- | --- | --- | --- |
| Uranium | 687.68 nM | 8.36 nM | 98.78 |
| Calcium | 7.00 mM | 6.54 mM | 6.60 |

**REFERENCES**

(1) Jøssang, T., Feder, J., Rosenqvist & E. Photon correlation spectroscopy of human IgG. *J. Protein. Chem.* **7 (2)**, 125-171 (1988).

(2) Melton, S. J., Yu, H., Williams, K. H., Morris, S. A., Long, P. E. & Blake, D. A. Field-Based Detection and Monitoring of Uranium in Contaminated Groundwater using Two Immunosensors. *Environ. Sci. Technol*. **43(17)**, 6703-6709 (2009).

(3) Blake, D. A.; Pavlov, A. R.; Yu, H.; Kohsraviani, M.; Ensley, H. E. & Blake, R. C. Antibodies and antibody-based assays for hexavalent uranium. *Analytica Chimica Acta* **444**, 3-11 (2001).

(4) Sahiner, N., Yu, H., Tan, G., He, J., John V. T. & Blake, D. A. Highly Porous Acrylonitrile-Based Submicron Particles for UO_2_^2+^ absorption in an immunosensor assay *ACS Appl. Mater. Interfaces* **4 (1)**, 163–170 (2012).

(5) Blake II, R. C.; Pavlov, A. R.; Khosraviani, M.; Ensley, H. E.; Kiefer, G. E.; Yu, H.; Li, X.; Blake, D. A., Novel monoclonal antibodies with specificity for chelated uranium(VI): isolation and binding properties. *Bioconjug Chem* ***15* (5)**, 1125-36. (2004).
